# Challenges to diagnose metaplastic carcinoma of the breast through cytologic methods: an eight-case series

**DOI:** 10.1186/1746-1596-6-7

**Published:** 2011-01-18

**Authors:** Seema Lale, Kiyoe Kure, Daniel Lingamfelter

**Affiliations:** 1 Department of Pathology, Albert Einstein College of Medicine at Long Island Jewish Medical Center, New Hyde Park, New York, USA; 2 Department of Pathology, Woodhill Medical and Mental Health Center, New York University School of Medicine, Brooklyn, New York, USA; 3 El Paso County Coroner's Office, Colorado Springs, Colorado, USA

## Abstract

Because metaplastic carcinoma of the breast encompasses a great variety of histopathology, diagnostic challenges abound, especially within the realm of cytology. The authors compiled and studied an eight-case series comprised of metaplastic breast carcinomas and lesions initially suspicious cytologically for metaplastic carcinoma in order to assess the degree of cytologic-histologic correlation and to identify recurring problematic themes surrounding the cytology-based diagnosis of this neoplasm.

The cytologic and histologic slides from eight cases suspicious for metaplastic breast carcinoma diagnosed by fine needle aspiration (FNA) were collected and analyzed through a seven-year retrospective search of case files at our institution. Based on cytologic characteristics, the cases were separated into three groups. Group 1 consisted of three cases presenting with poorly differentiated adenocarcinoma and squamoid components on FNA. Group 2 was composed of two cases that featured a monophasic, malignant ductal cell population on cytology, while the cytologic specimens for the third group of cases presented with a mesenchymal component with or without a malignant glandular constituent.

Cytologic-histologic correlation was present in two of three cases demonstrating a mesenchymal component, and there was 100% sensitivity in the cytologic detection of those mesenchymal elements. However, in only one of three cases was there an accurate cytologic diagnosis of metaplastic carcinoma when squamoid changes were present on FNA. Both cases demonstrating only malignant glandular elements on cytologic specimens revealed an additional component of malignant squamous differentiation upon the examination of mastectomy-derived tissue.

These results indicate that squamous-like changes identified on FNA should be interpreted with caution and that sampling error remains a problematic recurrence in cytology. Regardless, there appears to be promise concerning the accurate cytologic diagnosis of metaplastic carcinoma when the lesion is characterized by a mesenchymal component. A study implementing a larger case number is essential in determining the significance of these findings.

## Background

Metaplastic carcinoma refers to a highly heterogeneous group of neoplasms characterized by an admixture of adenocarcinoma with "metaplastic" areas typically of spindle, squamous, osseous, or chondroid differentiation. These lesions are rare, with a reported incidence of less than 1% of all breast tumors in some series [1]. Wargotz et al. suggested five variants of metaplastic carcinoma - matrix-producing carcinoma, squamous cell carcinoma, spindle cell carcinoma, carcinosarcoma, and metaplastic carcinoma with osteoclastic giant cells [2-7]. This entity known as "metaplastic carcinoma" can pose a diagnostic challenge, in part, because it encompasses a wealth of histopathologic variation and, consequently, can be mimicked by a wide array of other disease entities. We report a series of eight cases of metaplastic breast carcinomas or lesions initially suspicious cytologically for metaplastic carcinoma of the breast, describe their cytologic and subsequent histologic features, and discuss various challenges that we encountered in the cytologic-based diagnosis of such an entity.

## Case Presentations

Eight cases suspicious for metaplastic carcinoma of the breast diagnosed on fine needle aspiration (FNA) cytology were selected from the pathology files of Truman Medical Center, Kansas City, Missouri, from 2001 through 2008. Clinically, the lesions presented as localized masses and/or abnormal mammograms. Biopsy and subsequent mastectomy specimens were reviewed for correlation (or lack thereof) with the FNA results. All specimens were reviewed by two pathologists for uniformity of reporting. Clinical data was obtained by chart review.

## Materials and methods

Mammography studies were reviewed. The mammographic studies were reported using the Breast Imaging Reporting and Data System ( BI- RADS), which includes the following categories: BIRADS 1( negative), BIRADS 2 (benign), BIRADS 3 (probably benign), BIRADS 4 (suspicious abnormality), and BIRADS 5 (highly suggestive of malignancy).

The fine needle aspirations were performed using 22-gauge needles and three passes. Half of the smears were air-dried and half were fixed in alcohol. Air-dried smears were stained with Diff-Quik while alcohol-fixed smears underwent Papanicolaou staining. The smears were assessed for adequacy by immediately evaluating the Diff-Quik stained preparations.

The resected tissue was fixed in formalin, processed in a routine fashion, then sectioned and stained with hematoxylin and eosin. For the immunohistochemical analysis, 4-μm-thick paraffin-embedded tissue sections were deparaffinized and endogenous peroxidase quenched using 3% hydrogen peroxide in methanol. The sections were then hydrated through phosphate-buffered saline. For antigen retrieval, slides were placed in "target antigen retrieval" solution (DAKO Cytomation, Carpinteria, CA) and submerged into pre-heated antigen retrieval solution for 30 minutes in a Black and Decker steamer, after which slides were left sitting for ten minutes at room temperature, washed in phosphate buffered saline, and then exposed to the following antibodies and conditions for one hour: S-100 (1:100 dilution), cytokeratin (1:100 dilution) and CEA (1:2000 dilution). Blocking solution was used as a negative control on duplicate slides. Secondary antibody was biotinylated goat anti-immunoglobulin of mouse, rabbit, guinea pig, and rat primary antibodies (Supersensitive Immunodetection System, Biogenex, San Ramon, CA). An avidin-biotin-peroxidase complex with DAB as a chromogen was used for detecting antibody binding.

## Results

A total of 8 cases suspicious for metaplastic carcinoma of the breast diagnosed on fine needle aspiration were studied by a retrospective search of files over a period of 7 years. A retrospective review of the biopsies and mastectomies performed in each case was performed. The series consisted of 8 female patients ranging from 41 to 77 years of age, with a mean age of 59 years.

Grossly, tumor sizes ranged from 2.0 cm to 13 cm. Most tumors were described as gray, white and firm. One case contained areas of obvious necrosis.

Based on the fine needle aspiration findings, we divided the eight cases into three groups. Group 1 included three cases characterized by poorly differentiated adenocarcinoma and squamoid components on fine needle aspiration. In the first case, fine needle aspiration cytology revealed poorly differentiated ductal carcinoma with focal malignant squamous differentiation. Necrotic debris was also present. Subsequent biopsy and mastectomy findings coincided with the FNA findings, showing poorly differentiated ductal carcinoma with focal malignant squamous differentiation, consistent with metaplastic carcinoma of the breast. Estrogen receptor, progesterone receptor and Her2/neu receptor status were negative.

**Figure 1:**
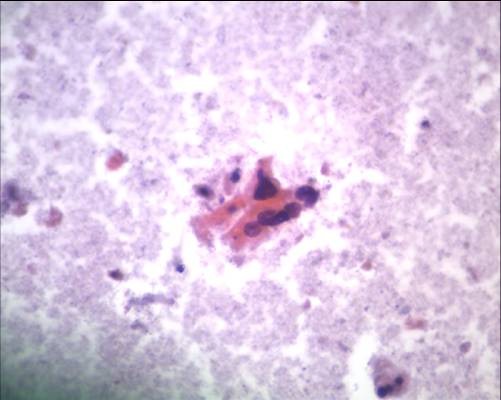
**Clusters of malignant cells with eosinophilic cytoplasm and dark, angulated nuclei resembling squamous cells (Papanicolaou, ×20)**.

**Figure 2:**
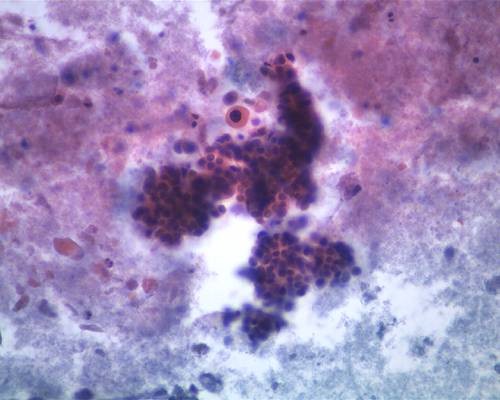
**A single, atypical squamoid cell displaying eosinophilic cytoplasm and a dark nucleus, next to a cluster of malignant glandular cells (Papanicolaou, ×20)**.

**Figure 3:**
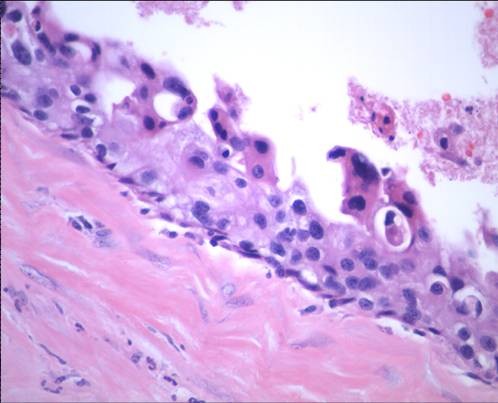
**Mastectomy specimen reveals microinvasive and in situ ductal carcinoma; no squamous component is seen (H&E, ×20)**.

Cytology in the second case in this group revealed multiple clusters of malignant ductal cells in a background of necrotic debris. One slide also revealed multiple clusters of large cell pearls. Single bizarre cells consistent with squamous cell carcinoma, mixed with ductal cells, were noted (Figures 1, 2). Additionally, clusters of ductal cells with apocrine metaplasia were identified. Interestingly, a subsequent mastectomy specimen revealed microinvasive and in situ ductal carcinoma (Figure 3). A comedo portion was identified and displayed abundant areas of necrosis lined by large hyperchromatic cells with thick eosinophilic cytoplasm. Squamous elements were not seen. The nipple areola complex was positive for Paget's disease.

The third case in this group revealed numerous groups of poorly differentiated malignant cells showing ductal and squamous differentiation, and subsequent biopsy revealed poorly differentiated invasive adenosquamous carcinoma. The tumor cells were focally positive for EMA but did not stain positively for synaptophysin, chromogranin, CK7, CK20, TTF-1, GCDFP, P63 or type IV collagen. Immunoperoxidase stains for ER, PR and Her-2/neu were negative. This immunohistochemical pattern ruled out a primary breast tumor. Instead, metastatic adenocarcinoma of unknown primary origin was diagnosed.

Group 2 included two cases that were cytologically monophasic. In the first case, touch preparations were performed and revealed poorly differentiated adenocarcinoma. Subsequent mastectomy revealed invasive, poorly differentiated adenocarcinoma with a malignant squamous population. In case 2, fine needle aspiration cytology also revealed only poorly differentiated adenocarcinoma. The subsequent mastectomy, like case 1, revealed invasive poorly differentiated adenocarcinoma admixed with malignant squamous cells. In both of these cases, squamous cells were not detected on touch preparations or fine needle aspiration, and diagnoses of metaplastic carcinoma were made only after examination of the mastectomy tissue specimens.

Group 3 included three cases, all of which cytologically revealed a prominent mesenchymal component (spindle or sarcomatoid population) with or without an adenocarcinoma component.

**Figure 4:**
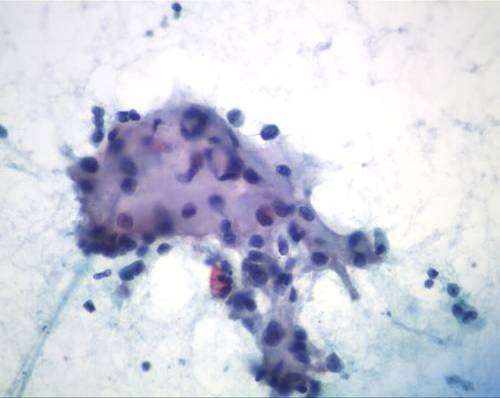
**Clusters of stromal cells admixed with malignant cells (Papanicolaou, ×20)**.

**Figure 5:**
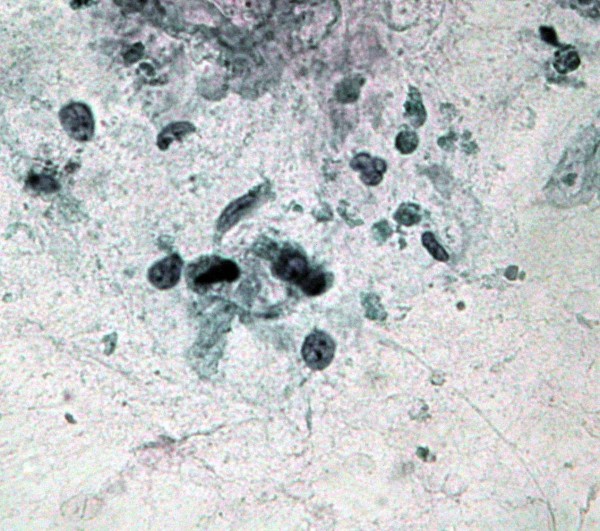
**Singled spindle cells and atypical cells with striped cytoplasm (Papanicolaou, ×20)**.

**Figure 6:**
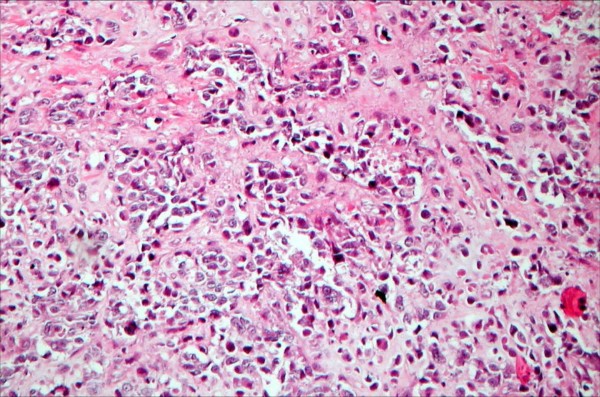
**Poorly differentiated invasive ductal carcinoma with sarcomatous changes (H&E, ×20)**.

Fine needle aspiration cytology in the first case revealed three-dimensional clusters and single malignant cells consistent with adenocarcinoma. A prominent component of malignant spindle cells was also identified. Tissue fragments of dense fibrous stroma admixed with malignant cells were observed (Figures 4, 5). The diagnostic impression was poorly differentiated adenocarcinoma, most likely of ductal origin, with possibly another poorly differentiated spindle cell component. Subsequent modified radical mastectomy revealed features of poorly differentiated invasive ductal carcinoma with sarcomatous changes (Figure 6), consistent with metaplastic carcinoma. Extensive areas of necrosis with focal chondroid differentiation were noted. Vascular invasion was present. This tumor was negative for progesterone and Her2-neu receptors but positive for estrogen.

During the FNA in case 2, 60 ml of cystic fluid was drained. Smears from the cyst wall showed atypical epithelioid and spindle cells in a background of fat necrosis, raising the suspicion for metaplastic carcinoma of the breast. The smears had high overall cellularity including multiple, scattered islands within a varying background of fat necrosis and a fibrillary, metachromatic stroma. Several mitotic figures were identified, but necrosis was absent. The case was diagnosed as suspicious for malignancy, with metaplastic carcinoma of the breast in the differential diagnosis. Subsequent biopsy and mastectomy revealed irregular, dense proliferations of plump spindle cells with moderate cytologic and nuclear pleomorphism. The majority of the nuclei showed prominent nucleoli. Mitotic figures were frequent. Additionally, tissue from the biopsy cavity demonstrated scattered, spotty foci of necrosis as well as both perivascular and perineural invasion by the tumor cells.

The proliferating spindle cells revealed diffuse positivity for the myoepithelial markers smooth muscle actin, CD10, and p63, and focal positivity for S-100. Vimentin positivity was strong and diffuse. Additionally, increased positivity for Ki-67 provided evidence for a high proliferation index among the neoplastic cells. The immunohistochemical markers glial fibrillary acid protein (GFAP), desmin, estrogen receptor (ER), and progesterone receptor (PR) did not highlight cells from the lesion. Last, positive staining with the pancytokeratin marker [AE1/AE3 + 8/18] ruled out the possibility of a sarcoma with myoepithelial differentiation. The histology and accompanying immunohistochemical staining patterns were consistent with an infiltrating myoepithelial carcinoma (i.e., sarcomatoid metaplastic carcinoma) of the breast.

**Figure 7:**
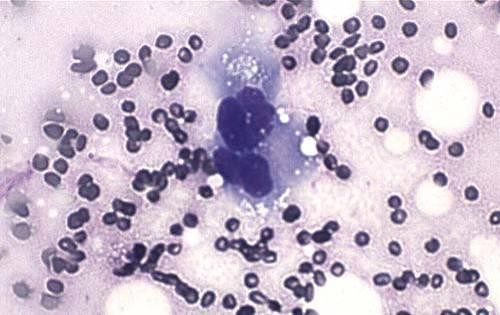
**Multinucleated giant cell (Diff-Quick, ×40)**.

**Figure 8:**
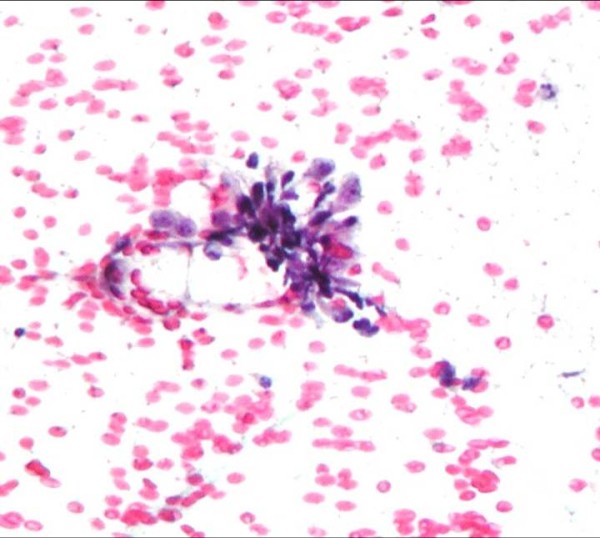
**Loosely arranged cluster of pleomorphic sarcomatoid cells (Papanicolaou, ×20)**.

**Figure 9:**
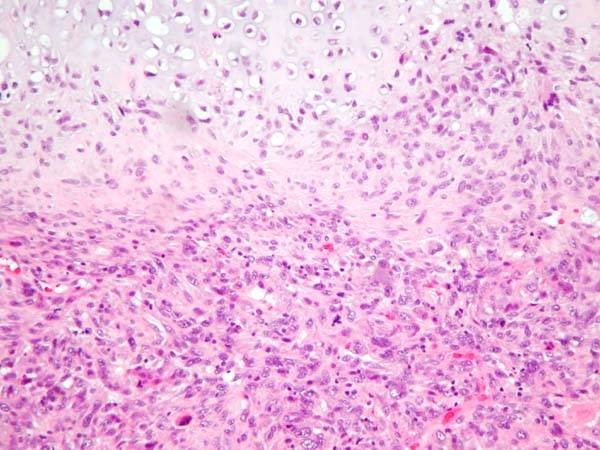
**Histologic features of the chondrosarcomatous and pleomorphic cell components (H&E, ×20)**.

Case 3 of this group showed large, pleomorphic sarcomatoid cells with bluish cytoplasm and few multinucleated giant cells and lymphocytes on cytologic smear specimens (Figures 7, 8). The case was diagnosed as favoring metaplastic carcinoma. Subsequent biopsy revealed chondrosarcomatous and pleomorphic spindle cell components with multinucleated giant cells and a brisk mitotic rate. Vimentin was positive in the chondrocytic cells while smooth muscle actin was positive in the spindle cells. S-100 and high molecular weight keratin (HMWK) were both negative. No epithelial component was identified. The differential diagnosis included chondrosarcoma and metaplastic breast carcinoma. A subsequent mastectomy specimen revealed a well-delineated tumor with a distinct chondromyxoid matrix composed of a cartilaginous component and a high grade noncartilaginous sarcoma (Figure 9). The tumor entrapped breast ductal structures. The noncartilaginous sarcoma was composed of highly anaplastic spindle cells with a slightly epithelioid appearance, hyperchromatic nuclei, prominent nucleoli, brisk mitoses (many abnormal) of approximately 11 per 10 high power fields, multinucleated tumor giant cells and osteoclast-type multinucleated giant cells. Interspersed among the high grade sarcomatous areas were nodular and lobulated islands of well-differentiated cartilage showing lacunar spaces occupied by pleomorphic chondrocytes with hyperchromatic nuclei, some of which were binucleated. The transition between the cartilaginous and sarcomatous components was sharp and abrupt without morphologic continuity. In addition there were strips of woven bone formation. S-100 immunostaining highlighted a few chondrocytes, and pancytokeratin (CK) was positive in breast ductal structures. Extraskeletal dedifferentiated chondrosarcoma served as the final diagnosis.

## Discussion

Metaplastic carcinomas of the breast comprise a rare, heterogeneous group of neoplasms with varying patterns of metaplasia and differentiation. The common feature of these tumors is the presence of a predominant component other than a glandular cell population. The "metaplastic" terminology applied to this entity refers to the transformation of the glandular component into another cell type, whether it be squamous cells or mesenchymal elements such as spindle cells and osteoclastic-like giant cells. These transformed cells may appear either benign or malignant.

Among our 8 cases, three (Group 1) showed squamous-like cells in addition to malignant glandular elements on FNA. Of the three, only case 1 had a perfect cytologic-histologic correlation. The cytology in case 2 showed large cells with bizarre nuclei and thick "keratinizing" cytoplasm, as well as what was originally thought to be keratin pearls. Surprisingly, the tissue from the mastectomy specimen revealed only microinvasive and in situ ductal carcinoma but also showed a comedo component containing cells characterized by dense, eosinophilic cytoplasm that strongly mimicked squamous cells on cytology. In our third case, poorly differentiated adenocarcinoma and squamous cells were revealed on fine needle aspiration, suggesting a possible metaplastic carcinoma. Biopsy confirmed the presence of both malignant glandular and squamous populations, consistent with adenosquamous carcinoma. However, immunohistochemistry ultimately ruled out the possibility of a primary breast tumor, leading to a diagnosis of metastasis.

Needless to say, care must be taken when squamoid changes are seen on FNA.

If we see suspected squamous cells on fine needle cytology, a broad differential diagnosis should be considered in addition to metaplastic carcinoma, including but not limited to such lesions as sarcoma with radiation-induced atypia, adenosquamous carcinoma, and any entity with metaplastic changes such as phylloides tumor or, as we encountered, ductal carcinoma in situ [8]. And, as case 3 shows, there is always the possibility of a metastatic lesion.

In the two cases comprising Group 2, only one component (malignant glandular) was found in the aspirates and touch preparations. However, subsequent histology revealed invasive adenocarcinoma with malignant squamous elements (i.e., metaplastic carcinoma). In our experience, 'sampling' impacts the fine needle aspiration diagnosis. Multiple needle passes and sampling of all areas of the lesion are ideal to ensure an accurate cytologic diagnosis, but in reality compromises oftentimes must be made in response to patient comfort. Therefore, the pathologist commonly must settle for suboptimal sampling -- too few passes, or perhaps sampling areas representing less than 50% or even 25% of the lesion. Also, metaplastic carcinomas not uncommonly reveal prominent cystic or necrotic areas, contributing to further complications in the identification of an additional cell population, especially when less than optimal sampling has occurred [9].

Two of the three cases from the third "mesenchymal" group showed perfect cytologic-histologic correlation, and the mesenchymal components in all three cases were detected on cytology. FNA specimens from case 1 contained both ductal and sarcomatous components of the tumor, while the cytologic examination in case 2 was positive for both epithelioid and spindle cell constituents of the myoepithelial carcinoma. An immunohistochemical panel for keratins was essential to the diagnostic workup of the latter case. Currently, no specific myoepithelial marker is available, so a battery of myogenic markers including basal cell type cytokeratins, p63, and S-100 was performed on subsequent biopsy specimens to establish a myoepithelial differentiation [10]. The cytology in the third case demonstrated large, sarcomatous cells and some multinucleated cells, thereby allowing for the detection of the neoplasm's mesenchymal derivation. Although no distinct epithelioid component was detected, a suspicion for metaplastic carcinoma nonetheless was raised. Primary chondrosarcoma, the eventual diagnosis for this lesion, is a very rare entity but should be kept in mind in the differential diagnosis in such cases, and this diagnosis should be confirmed by cytokeratin and EMA staining of the spindle cell component. In general, the differential diagnosis of spindle cell breast lesions includes metaplastic carcinoma, fibromatosis, pseudoangiomatous stromal hyperplasia, nodular fasciitis, inflammatory myofibroblastic tumor, phyllodes tumor with stromal overgrowth, and metastases [11]. Primary, pure sarcomas of the breast are very rare, the most common of which is malignant fibrous histocytoma, but these lesions still must be considered as possibilities.

## Conclusion

In conclusion, our eight-case series identified several cytologic themes that surfaced in the course of diagnosis. Cytologic specimens were 100% sensitive (3/3) in the detection of mesenchymal elements, regardless of the fact that one of these cases ultimately was not diagnosed as metaplastic carcinoma. On the contrary, only in one of our cases was metaplastic carcinoma accurately diagnosed when squamoid changes were present, apparently as a result of a squamous cell mimicker and an unusual metastasis. Lastly, the two cases that were cytologically monophasic for malignant glandular elements both revealed malignant squamous components on subsequent mastectomy-derived tissue, raising speculation that sampling error remains a common culprit in cytology. This series presents intriguing findings, but our case number is quite limited. A significantly larger case series therefore would be beneficial to determine if such diagnostic themes persist on a grander scale.

It is prudent that pathologists and clinicians understand not only the benefits but also the limitations of cytologic diagnoses from FNA specimens. Immediate evaluation of specimen adequacy is useful to eliminate equivocal diagnoses from technical factors. Combining findings derived from both cytology and histology best allows for the proper management of each patient.

## Consent

Consent was obtained for publication of this case report and accompanying images. A copy of the consent is available for review by the Editor-in-Chief of this journal.

## Competing interests

The authors declare that they have no competing interests.

## Authors' contributions

SL and DL constructed the majority of the manuscript. SL and KK were responsible for cytology and histology interpretations. DL and KK contributed significantly to manuscript proofreading and revisions. All authors read and approved the final manuscript.
